# DNA Methylation Assessed by SMRT Sequencing Is Linked to Mutations in *Neisseria meningitidis* Isolates

**DOI:** 10.1371/journal.pone.0144612

**Published:** 2015-12-11

**Authors:** Mohamad R. Abdul Sater, Araceli Lamelas, Guilin Wang, Tyson A. Clark, Katharina Röltgen, Shrikant Mane, Jonas Korlach, Gerd Pluschke, Christoph D. Schmid

**Affiliations:** 1 Swiss Tropical and Public Health Institute, Socinstrasse 57, Basel, Switzerland; 2 Universität Basel, Petersplatz 1, Basel, Switzerland; 3 Yale Center for Genomic Analysis, Yale University, New Haven, Connecticut, United States of America; 4 Pacific Biosciences, Menlo Park, California, United States of America; 5 SIB Swiss Institute of Bioinformatics, Basel, Switzerland; Georgia Institute of Technology, UNITED STATES

## Abstract

The Gram-negative bacterium *Neisseria meningitidis* features extensive genetic variability. To present, proposed virulence genotypes are also detected in isolates from asymptomatic carriers, indicating more complex mechanisms underlying variable colonization modes of *N*. *meningitidis*.

We applied the Single Molecule, Real-Time (SMRT) sequencing method from Pacific Biosciences to assess the genome-wide DNA modification profiles of two genetically related *N*. *meningitidis* strains, both of serogroup A. The resulting DNA methylomes revealed clear divergences, represented by the detection of shared and of strain-specific DNA methylation target motifs. The positional distribution of these methylated target sites within the genomic sequences displayed clear biases, which suggest a functional role of DNA methylation related to the regulation of genes.

DNA methylation in *N*. *meningitidis* has a likely underestimated potential for variability, as evidenced by a careful analysis of the ORF status of a panel of confirmed and predicted DNA methyltransferase genes in an extended collection of *N*. *meningitidis* strains of serogroup A. Based on high coverage short sequence reads, we find phase variability as a major contributor to the variability in DNA methylation. Taking into account the phase variable loci, the inferred functional status of DNA methyltransferase genes matched the observed methylation profiles.

Towards an elucidation of presently incompletely characterized functional consequences of DNA methylation in *N*. *meningitidis*, we reveal a prominent colocalization of methylated bases with Single Nucleotide Polymorphisms (SNPs) detected within our genomic sequence collection. As a novel observation we report increased mutability also at 6mA methylated nucleotides, complementing mutational hotspots previously described at 5mC methylated nucleotides.

These findings suggest a more diverse role of DNA methylation and Restriction-Modification (RM) systems in the evolution of prokaryotic genomes.

## Introduction

### *Neisseria*: pathogenicity and genomic plasticity

*Neisseria meningitidis* is a commensal Gram-negative bacterium exclusively found in the human nasopharyngeal mucosa and is readily transmitted via respiratory secretions or saliva [1]. A small proportion of individuals colonized by a virulent strain may develop invasive disease including sepsis or meningitis [2], especially devastating as epidemics in the African ‘meningitis belt’ [3]. Regular transmission events in meningitis outbreaks indicate that the invasive colonization mode is at least in part 'inheritable', in other words a bacterial population can maintain its disease causing phenotype. However not all transmissions necessarily lead to disease, a complex interplay of host-pathogen interactions influences the outcome of invasive infections [4]. Vaccination projects have dramatically lowered the incidence of meningococcal disease, yet the asymptomatic carriage and the high genetic variability of meningococci might be responsible for occasional reemergence of epidemics [5]. Genome sequencing of steadily increasing numbers of *N*. *meningitidis* strains revealed substantial homologous recombination with DNA cleavage mechanism suggested to be associated with phylogenetic clades [6]. Frequent horizontal transfer of DNA elements as well as a range of genetic variation mechanisms in *N*. *meningitidis* including phase variation [7] require extra caution in the interpretation of nucleotide changes. A number of genotypes were suggested to be associated with virulence including genes involved in the synthesis of the polysaccharide capsule. Yet to present no strict pathogenic genotype is defined which would allow to distinguish disease-causing strains from avirulent carrier strains [8].

### Prokaryotic epigenetics and detection of DNA modifications using SMRT

In eukaryotes, epigenetics has emerged as a significant phenotypic determinant representing an additional layer to the sequence of nucleotides in a genome, as showcased by the epigenetic roadmap project [9]. DNA methylation in prokaryotes differs by more diverse modification types including N6-methyladenine (6mA), N4-methylcytosine (4mC) and 5-methylcytosine (5mC), deposited by a diverse set of methyltransferases at specific target sequences (motifs). Prokaryotic DNA methylation is therefore not concentrated to the CpG dinucleotide context and was in the past mainly characterized as part of Restriction-Modification (R-M) systems and its antiviral defense mechanisms cleaving any unmodified ‘non-self’ DNA [10]. Contemporary sequencing methods determine genome-wide epigenetic DNA modification maps. Pacific Biosciences' Single Molecule, Real-Time (SMRT) sequencing method is based on the direct monitoring of the processing of single DNA molecules by DNA polymerase [11]. The kinetics of DNA synthesis enables the genome-wide determination of diverse DNA modifications [12], which represents a unique advantage for studying prokaryotic epigenetics [13]. The approach has previously been successfully applied to the genome-wide mapping of methylated adenine and cytosine residues in multiple organisms including pathogenic *Escherichia coli* [14], *Helicobacter pylori* [15], *Caulobacter crescentus* [16], *Mycoplasma* [17] and *N*. *meningitidis* [18]. SMRT sequencing enabled to determine previously unknown target sequences and the exact site of methylation of specific methyltransferases [19]. Yet these experiments revealed also considerable divergence in the target sequences and/or methylation efficiency, if comparing homologous alleles of methylation enzymes in related strains differing by only a few amino acids [20].

A number of studies in diverse prokaryotic systems have linked deficiencies in DNA methylation with altered gene expression patterns [21], [22], [20], [23]. However the molecular mechanisms for direct effects of DNA methylation on prokaryotic gene expression are presently not elucidated, and only in single cases for instance a positional overlap of differentially methylated target sites with binding sites of transcription factors could be shown [24] [16]. In many cases the detected methylation sites can not be directly linked to a larger number of differentially expressed genes. Accordingly alternative molecular effects of DNA methylation are proposed, including interactions at the origin of replication and an involvement in genome replication [25].

Variable DNA methylation in *Neisseria* species has been reported previously [26], yet no direct association of the activity of a specific DNA adenine methyltransferase (Dam) with virulence was found [27]. More recently different alleles of the mod DNA methyltransferase gene family undergoing phase variability were associated to divergent cellular phenotypes [21].

Given the described variability in genomic sequences and phenotypes, we set out to investigate the epigenetic DNA modification profiles in *N*. *meningitidis* isolates. We determine DNA methylation target motifs (one or several DNA sequences), and our analysis reveals biased distributions of these target sequences in the genomes. We observe high variability in the methylation profiles among a population of closely related bacterial isolates. Strikingly, we also discover enrichments of SNPs at the precise positions of methylated bases in the genomes, pointing to a role of DNA methylation in the evolution of favorable genome configurations.

## Materials and Methods

### Cultivation of strains of N. meningitidis, isolation of genomic DNA

*Neisseria meningitidis* reference strain Z2491 (DSM No. 15465) was obtained from DSMZ (Braunschweig, Germany). *N*. *meningitidis* isolates were previously collected over a time period of ~10 years during meningococcal meningitis epidemics in Sub-Saharan Africa (two sequence types ST2859 and ST7). Isolates underwent typically 2 rounds of single colony sub-culturing and over-night expansion *in vitro*. For genomic DNA preparation, strains were grown on supplemented GC agar base (Oxoid) plates for 20–24 hours in 5% CO_2_ at 37°C. Single colonies were transferred into liquid Brain Heart Infusion (BactoTM) medium and again incubated overnight in 5% CO_2_ at 37°C. Genomic DNA was extracted as described previously [28]. Genomic DNA samples of two strains (isolate NM1264 and reference Z2491) were subjected to SMRT sequencing. The material of strain NM1264 represented aliquots of a genomic DNA sample previously subjected to the Illumina sequencing method [29].

### Methylation sensitive restriction digest

NlaIV restriction enzymes (methylation sensitive target sequence GGNNCC) were obtained from New England Biolabs (catalog #R0126) and used according to manufacturer specifications to digest 1 ug of genomic DNA of each strain.

### SMRT sequencing

Genomic DNA preparations were sheared by sonication to ~500bp fragments, aiming at shorter reads with an increased coverage for DNA modification detection. To enhance detection of 5mC modifications, enzymatic conversion of 5- methylcytosine (5mC) to 5-carboxylcytosine (5caC) was carried out using the 5mC Tet1 oxidation kit (WiseGene) with an input of ~500ng of genomic DNA [30]. Generation of SMRTbell libraries and SMRT sequencing were performed following manufacturer instructions [31] to obtain a strand-specific sequencing coverage of about 50X on a standard PacBio RS instrument at the Yale Center for Genomic Analysis. Sequencing reads were aligned to Z2491 reference genome (AL157959), or to the genome assembly of strain NM1264 (344 contigs in S4 Text). To identify modified positions, we used Pacific Biosciences’ SMRTPortal analysis platform, v. 1.3.1. In brief, at each genomic position, modification scores (modQV) were computed as the -10 log of a p value for representing a modified base position, based on the distributions of the kinetics of base incorporation (IPD ratios) from all reads covering this position and from *in silico* kinetic reference values (details are available at http://www.pacb.com/pdf/TN_Detecting_DNA_Base_Modifications.pdf, [32]). Methylated sequence motifs were identified as previously described [20]. The SMRT sequencing data that were used in this paper have been submitted to the ENA databases under accession No. PRJEB11526.

### Local deviations in positional distributions of methylation motifs

Occurrences of methylation target sequences in genome sequences were determined using the fetchGWI tool [33]. The start positions and orientations of 1997 annotated ORFs [34] were used as 'reference feature' to sum up the occurrence counts for each methylation target motif ('target feature') using the ChIP-Cor tool (http://ccg.vital-it.ch/chipseq/chip_cor.php). Thereby motif counts were aggregated within 50bp windows positioned relative to the start (position zero) of each ORF. Statistical significance for the observed depletions/enrichments in the plotted counts was derived from a comparison to 1000 sets of simulated reference features with 2000 random genomic loci each. P values represent the fraction of random reference feature sets exhibiting aggregate motif counts across their corresponding 50bp windows more extreme than the count observed across the 50bp windows of the ORF set.

### Identification of DNA methyltransferase genes

Protein sequences of methyltransferases as obtained from REBASE (*rebase*.*neb*.*com*) were used to identify genes with >80% identity via BLAST searches. Potential methyltransferase ORFs were attributed the REBASE annotation, as available for the reference strain Z2491. For each of our isolate strains each methyltransferase ORF was verified for indels and SNPs (see SNP calling below) altering the frame or introducing premature stop mutations and thereby deactivating the enzyme.

### SNP calling and determination of recombination fragments

Single Nucleotide Polymorphisms (SNP) detection was performed as described in [29].In brief, paired-end Illumina reads (sequence data available at http://www.sanger.ac.uk/resources/downloads/bacteria/neisseria.html#project_1893) were mapped against the genome sequence of the *N*. *meningitidis* serogroup A, ST4 strain Z2491 [34] using SMALT version 0.7.4. Candidate SNPs were identified using SAMtools mpileup as previously described [35], with subsequent quality filtering (score≥30). Repetitive regions (>50bp) on the reference genome were identified using repeat-match [36], and MUMmer [37] to exclude SNPs called in repetitive regions. The iterative algorithm Gubbins was applied using default parameters to identify the recombination fragments [38]. Gubbins identifies recombinant fragments in collections of related genome based on elevated densities of base substitutions suggestive of horizontal sequence transfer, compared to frequencies of base substitutions in non-recombinant regions estimated from a maximum likelihood phylogeny.

### Co-occurrence of SNPs at methylation motifs

Based on coordinates in BED format of SNPs and of individual bases within target motifs (or non-target control motifs), we determined the number of overlapping positions using the intersect and count commands of BEDTools [39]. For plotting, the overlap counts between mutated bases and methylation sites were normalized by the number of genome wide motif occurrences and multiplied by a scaling factor x1000. The specificity of the overlaps to methylated positions was ascertained by the comparison to unmethylated positions within methylation target sites, as well as within 2 similar control sequences not known as DNA-methylation targets. To test the statistical significance of the observed increased overlaps, we assumed a random distribution of SNPs over the genome. The null hypothesis of independence between mutations and methylations was tested using the Chi-square approximation to the hypergeometric distribution

## Results

### SMRT sequencing determines divergent DNA modification profiles

We assayed the DNA methylation profiles of 2 *N*. *meningitidis* strains, selecting NM1264 and its closest reference strain Z2491 (both of serogroup A) for SMRT sequencing at a coverage for each strand approximating 50x on Tet1 converted genomic DNA samples.

**Fig 1:**
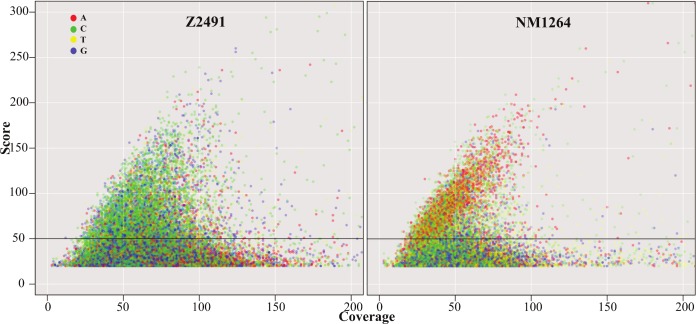
Two N. meningitidis serogroup A strains Z2491 and NM1264 display divergent DNA modifications. DNA modification scores are plotted against the coverage in SMRT sequencing of Tet1 converted samples. Each dot represents a position on either strand with a modification score larger than 20, the color specifying the nucleotide base, on which the modification was detected. Modified adenosines (red dots) are predominantly detected in strain NM1264. The horizontal line indicates the threshold score 50 applied for subsequent motif finding.

**Fig 2:**
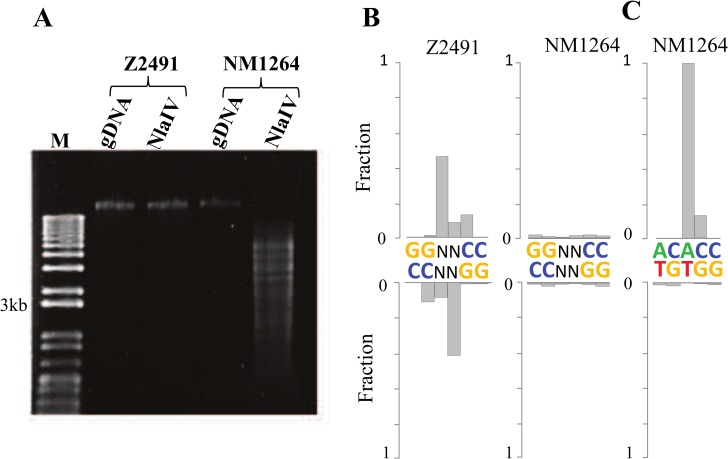
Methylation-sensitive restriction assays to validate DNA methylation derived from SMRT sequencing (A) DNA samples separated via gel electrophoresis, lane labels indicating (M) 1 kb Plus DNA Ladder (Invitrogen), (gDNA) whole genomic DNA preparations, and (NlaIV) restriction digest products targeting sites 'GGNNCC' in a methylation-sensitive manner. Samples from 2 different strains (Z2491 and NM1264, genome size: 2.18 Mb) display differential resistance consistent with DNA methylation profiles.(B) single position resolution of modification signal averaged over ~1800 'GGNNCC' sites in the respective genome assemblies. The fractions of sites exhibiting a modification score above 50 are displayed for each position and strand. (C) For comparison, adenosine methylation featuring enhanced sensitivity and positional resolution averaged over ~4000 'ACACC' sites.

The kinetics of polymerase extension steps were compared with previously recorded control values for highly similar, unmodified reference sequences [40]. We observed diverse kinetic variation signals, some of which could be attributed to known modification events such as DNA methylation. DNA methylation on each genomic position was represented by a probabilistic modification score (“modQV”) comprising base incorporation rates differing from that of the unmodified reference sequences. A genomic position is covered by several sequenced DNA fragments, and the modification scores include the consistency by which a specific modification was observed (S2 Text and S3 Text). SMRT sequencing assessed both DNA strands independently, accordingly we determined for strain Z2491 comparable average modification scores of 78.97 over 5237 sites with a modification score > 50 on the forward strand versus an average of 80.27 over 5246 sites on the reverse strand. In a plot of modification scores against sequencing coverage (Fig 1), both strains displayed a signal for modified cytosines (green dots). Spurious signals on non-cytosine bases in strain Z2491 are due to secondary peaks from nearby modified cytosines, presumably originating from a limited positional resolution of 5mC even after Tet1 conversion (see Fig 2B).

Modification scores on adenosine bases (red dots) were clearly dominant in strain NM1264. If comparing to SMRT sequencing of unmodified aliquots of identical DNA samples (S1 Fig), we find a satisfactory specificity of the Tet1-conversion for 5mC, with a minor reduction of the modification scores for 6mA.

In order to identify DNA recognition sequences of prokaryotic methyltransferases, we applied the SMRT® Analysis software suite from Pacific Biosciences to interpret the kinetic variation data on a genome-wide scale. We identified sequence motifs associated with a consistent kinetic variation pattern. Table 1 summarizes sequence motifs with a stringent modification score threshold >50.

To relate the discovered sequence motifs with information from REBASE [41] and the ORF status of the corresponding gene in the genome sequences, we assessed the presence of functional ORFs of DNA methyltransferase genes in the assembled genome sequences. We compiled a set of 13 DNA methyltransferase genes (R-M genes) occurring in our genomes (Z2491 and NM1264), based on sequence similarity with established DNA methyltransferase genes in all bacterial species in REBASE.

**Table 1:** Summary of detected modifications at DNA target motifs.

SMRT identified motifs		Closest motif in REBASE	GeneID / REBASE entry	ORF status	
Z2491	NM1264			Z2491	NM1264
C^**5m**^ **C**GG	C^**5m**^ **C**GG	C^**5m**^ **C**GG	M.NmeAI	ON	ON
T^**5m**^ **C**TGG	T^**5m**^ **C**TGG	T^**5m**^ **C**TGG	M.NmeAORF1035P	ON	ON
C^**5m**^ **C**WGG	C^**5m**^ **C**WGG	CCWGG	M.NmeAORF1500P	ON	ON
GGNN^**5m**^ **C**C	—	GGNNCC	M.NmeAORF1453P	ON	early stop
—	AC^**6m**^ **A**CC	—	*ModA12* M.NmeAORF1589P	early stop*	ON
—	—	—	*ModB2* M.NmeAORF1467P	early stop*	early stop*
—	ATGC^**6m**^ **A**T	ATGCAT	M.Nme2594ORF759P	not present	ON
—	—	CATG	M.NmeAORF59P	early stop	early stop
—	—	GGTGA	M.NmeAORF191P	early stop	early stop
—	—	GCCG^**6m**^ **A**G	RM.NmeAIII	early stop	early stop
—	—	[C/T/G]A	M.NmeAIV	ON	ON
—	—	—	M.NmeAORF1038P	early stop **	early stop**
—	—	—	M.NmeAORF1385P	early stop	early stop

DNA methylation target motifs as inferred from sites featuring a SMRT modification score >50. *N*. *meningitidis* strains Z2491 and NM1264 feature shared and strain-specific motifs, mostly with a correspondence in REBASE. Suffix 'P' in the GeneID specifies a methyl-transferase gene predicted by REBASE based on sequence homology. M.NmeAIV is reported to be normally inactive *in vivo*, possibly requiring additional factors for expression or activity.

* premature stop due to phase variable mutation

** premature stop codon in specificity subunit of Type I R-M system comprising M.NmeAORF1038P.

This comparison allowed attributing the identified motifs to established DNA methylation target motifs (Table 1). At least two DNA methylation motifs were identified to be common in both *N*. *meningitidis* strains. A sequence motif predicted in each strain fits the C^5m^ **C**GG target motif of the methyltransferase gene M.NmeAI active in both strains. Multiple partially overlapping motifs could be attributed to either the T^5m^CTGG target motif of M.NmeAORF1035P or to the related CC[AT]GG target motif of the methyltransferase gene M.NmeAORF1500P. Given the considerable similarity of these two target sequences including ambiguous positions, we can not completely exclude technical artifacts in our motif discovery defining the target sequence motifs, or incomplete sequence specificity descriptions in REBASE.

Two adenosine methylation motifs were detected exclusively in strain NM1264, consistent with the global DNA modification scores in Fig 1. The motif ATGC^6m^AT matches the (predicted) target sequence for M.Nme2594ORF759P in REBASE. The motif AC^6m^ **A**CC can be attributed to modA12 (M.NmeAORF1589P), which is the only remaining DNA methyltransferase with functional ORF solely in strain NM1264 (Table 1). This finding is in concordance with a recently published identification of specificities of Mod enzymes in *N*. *meningitidis* [18]. Target specificities of methyltransferases are generally inferred based on sequence similarity to closely homologous enzymes. For phase varion associated modA genes there is however a considerable diversity in DNA recognition domains reported specifically for pathogenic *Neisseria* [42]. Notably the target specificity of ModA12 reported here and elsewhere differs from the 5'-AGAAA-3' recognition site of a related modA13 allele in *N*. *gonorrhoeae* [21]. Our SMRT sequencing results resolved furthermore the position of the modified base within target sequences with a yet undetermined position as reported by REBASE, exemplified by ATGC^6m^AT for M.Nme2594ORF759P (Table 1). Given the still limited positional resolution of 5mC even after Tet1 conversion (see also Fig 2B), the position calls were considered particularly reliable for 6mA modifications.

Our SMRT sequencing detected a modification of the sequence motif GGNN^5m^C**C only in strain Z2491**, associated with the gene M.NmeAORF1453P featuring a complete ORF solely in the strain Z2491. The existence of a methylation-sensitive restriction enzyme NlaIV targeting an identical sequence motif (GGNNCC) allowed to validate the differential methylation as detected by SMRT sequencing. Accordingly NlaIV fragmented the genome of strain NM1264, whereas the Z2491 genome methylated at GGNNCC sites resisted NlaIV digestion (Fig 2A).

The results of these restriction digests indicated a complete protection and therefore a genome-wide methylation of 'GGNNCC' sequences in the strain Z2491. However only 48% of the 1817 instances of 'GGNNCC' sequences were called as modified in SMRT sequencing (Fig 2B). This limited sensitivity was presumably due to a very stringent threshold >50 for the SMRT modification score, to an incomplete enzymatic Tet1 conversion, and/or to limited positional precision of the kinetic signature of 5caC (Tet1-modified 5mC). In clear contrast, the fractions of modified bases were below 1% for the NlaIV restriction sensitive strain NM1264.

Spurious partial modifications of specific sequence motifs might derive from partial sequence overlaps with methylated target motifs. At least for the CCGG motif with 22% of detected modification signals, the partially overlapping GGNNCC target sequences appears not to significantly bias the fraction of detected modifications. Excluding the 358 instances of DDGGNNCCGG or CCGGNNCCHH sequences (D = not-C; H = not-G) in the Z2491 genome, we detected a SMRT modification signal at 19% of CCGG sequences.

Most of the discovered sequence motifs were palindromic, and accordingly a modification signal was also detected on the 'mirror' base on the opposite strand. The motif AC^6m^ **A**CC is exemplifying the strand-specificity and sensitivity of the SMRT sequencing on adenosine methylation, for this non-palindromic motif consequently no signal was observed on the opposite strand (Fig 2C). In conclusion, SMRT sequencing of two related *N*. *meningitidis* strains of serogroup A revealed divergent DNA methylation profiles associated with the functional status of DNA methyltransferase genes. In addition our approach enabled the confirmation of target motifs for predicted DNA methyltransferase genes in *N*. *meningitidis*.

### Methylation target motifs with biased distributions in regulatory genomic regions

**Fig 3:**
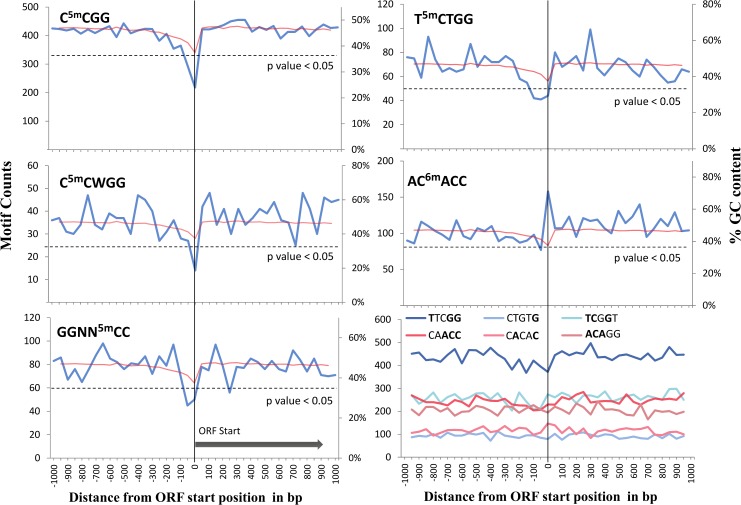
Depletion of cytosine methylated motifs in the immediate upstream region of ORFs. Occurrence counts of five (most frequent) methylation target motifs are plotted against their position (in bp) relative to 1997 oriented ORFs (genome annotation N. meningitidis strain Z2491). Motif counts are presented as sum over all ORFs within 50 bp windows, centered at position zero. Red lines in each panel compare to the GC content percentage (y-axis label to the right), averaged over all ORF regions. Dashed horizontal lines represent the averaged motif occurrences corresponding to statistically significant (p value 0.05) depletion of the corresponding motif, derived from a comparison to equally sized sets of random loci. The lower right panel represents occurrence counts of a set of six non-methylated control motifs with similar base composition (identical positions in bold), and similar occurrence frequencies as the non-palindromic target motifs T5mCTGG and AC6mACC. None of the control motifs display significant depletions at ORFs comparable to that of methylated motifs.

Functional consequences of DNA methylation are incompletely characterized. Moreover the genomic locations of DNA parts with regulatory functions are not precisely established in *N*. *meningitidis*. We therefore focused on sequences immediately upstream from genes, which were suggested to harbor a considerable proportion of loci under purifying selection based on the analysis of phylogenetically conserved sequences in prokaryotes [43]. We considered all sequences matching the methylation target motifs identified by SMRT, as an alternative to actual SMRT modification scores limited by the sensitivity and positional precision for 5mC modifications. We applied a cumulative analysis of the occurrence of methylation motifs relative to a set of 1997 start positions of annotated ORFs. The aggregation over a large set of loci renders this ChIP-cor analysis (see methods for details) very sensitive for recurring local deviations in linear distributions. At distances up to 1kb to ORF start positions, methylation motifs were detected at frequencies in general closely approaching the average genome wide frequencies (Fig 3). Only the motif occurrences immediately upstream from ORFs displayed a significant deviation (p value < 0.05), if compared to motif counts in equally sized sets of random loci. The observed deviations displayed a larger magnitude than the average GC content, which is only slightly decreased at the ORFs (Fig 3). To further control for base composition effects, we assessed the positional distributions of a set of non-methylated sequence motifs without overlaps with target motifs described in this study, with similar base composition as the two non-palindromic target motifs, and not specifying exclusively G and C bases. Unlike methylation target motifs, these control sequence motifs displayed no significant deviation, if compared to motif counts at random loci as described above.

We have extracted 120 ORFs displaying at least one AC^6m^ **A**CC motif within the interval from -75bp to their start position, but the current annotations of the large majority of those genes (hypothetical protein, unknown function) did not allow to identify particular functional groups sharing methylation target sequences in their regulatory sequences. An analogous analysis for each of the 5-methylcytosine motifs neither led to the identification of over-represented gene categories, functions or localization. Nevertheless the observed clear biases in the positional distribution of methylated target sites strongly suggests a functional role of DNA methylation likely related to the regulation of genes.

### Variable set of active DNA methyltransferase genes in *N*. *meningitidis* isolates of serogroup A

In order to establish the potential of DNA methylation in the genomes of a collection of *N*. *meningitidis* strains, we extended the assessment of the presence of functional ORFs of DNA methyltransferase genes to assembled genome sequences of 101 strains of *N*. *meningitidis* previously collected over a time period of ~10 years during meningococcal meningitis epidemics in Sub-Saharan Africa, clustering into two sequence types (ST2859 and ST7) [29]. We included two reference strains of serogroup A, namely WUE2594 [44] and Z2491 [34].

**Fig 4:**
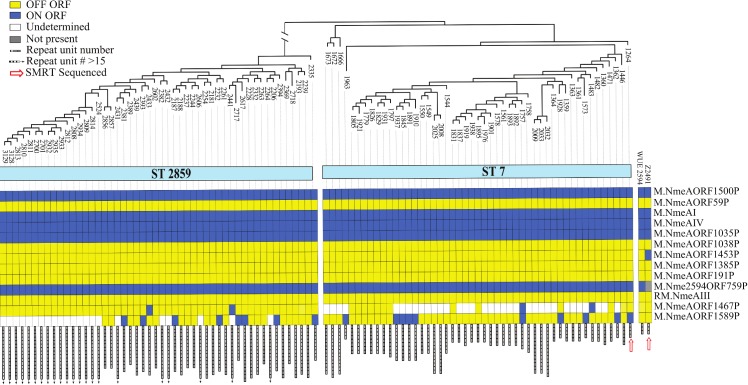
Variability at DNA methyltransferase loci. 101 N. meningitidis isolates clustered according to SNP distance, yielding in two sequence type (ST) groups. Each column represents an isolate and rows specify the ORF status of 13 DNA methyltransferases (Rebase geneIDs of Z2491 reference strain). Bars in grey at the bottom represent the number of repeat units determining ON/OFF status of the phase-variable modA12 (M.NmeAORF1589P)

Our analysis of the matrix of predicted DNA methylation activities revealed the genomic diversity within the 101 serogroup A strains assessed here. While the majority of DNA methyltransferase genes display constant presence/absence (ORF ON/OFF) patterns (Fig 4), selected genes featured a larger diversity than to be expected from the global genome sequence similarity. Contributing to the ON/OFF diversity, we detected point mutations leading to premature stop codons (M.NmeAORF1453P in all strains except Z2491). Deletion of complete genes (M.Nme2594ORF759P = NMAA_0759) are likely related to genome rearrangement events and horizontal gene transfers. The largest part of divergence between the strains is however due to phase variability in two Type III methyltransferase genes modB2 (M.NmeAORF1467P) and modA12 (M.NmeAORF1589P).

We used for SMRT sequencing an aliquot of the genomic DNA preparation of strain NM1264 previously subjected to the Illumina sequencing method. Thereby we detected only 198 sequence variants (S1 Text) if mapping circular consensus reads from SMRT sequencing at an average coverage of approximately 100x (twice 50x from each strand) to contigs assembled from Illumina reads (~300x coverage [29], S4 Text). Hence the augmented number of indels in individual sub-reads of the SMRT method are effectively averaged out if DNA fragments are read multiple times and unified into circular consensus sequences.

**Fig 5:**
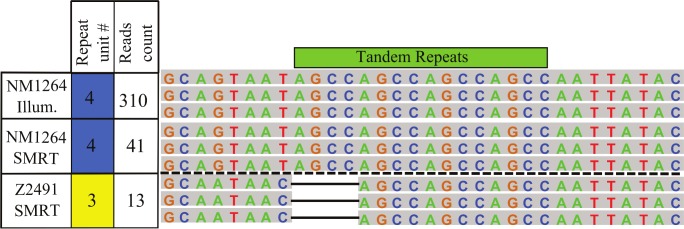
Phase variability at modA12 locus: Alignment of representative read sequences derived from genome sequencing (strains NM1264 or Z2491). The number of repeat units is indicated in the second column, with blue cell background indicating a resulting ON status of the ORF, and yellow for an OFF status. The third column indicates the total number of reads matching the corresponding repeat configuration.

As standard genome assembly and read mapping algorithms consistently failed especially at longer microsatellite repeat regions [45], we determined the repeat unit numbers directly from Illumina reads covering the corresponding locus (Fig 5). The determined repeat numbers enabled to call the ORF status at the ModA12 locus (ON: 18 strains; OFF: 59) and at the ModB*2* (ON: 4; OFF: 62). The read length of 75bp represented a limit to determine the number of microsatellite repeat units ('AGCC' for modA12 and 'TTGGG' for modB2) flanked by at least 5bp of non-repeat sequence. We could therefore not determine the ORF status at modA12 for 22 strains or at modB2 loci for 33 strains, respectively. These genomes contain in all likelihood repeats of a lengths exceeding the read length, for instance more than 15 x(AGCC) repeat units at the modA12 locus (Fig 4). Strikingly a few genomic DNA preparations yielded in a limited number of sequence reads containing repeat units divergent from the majority of reads covering the corresponding locus. Assuming no cross-contamination from other samples, these reads might be products of intra-clonal variability, consistent with increased mutations rates at phase variable loci [46]. In conclusion, our careful analysis of the ORF status of a panel of DNA methyltransferase genes revealed phase variability as a major contributor to variability in the DNA methylomes of isolates assessed here.

### Mutations over-represented at DNA methylation target motifs

We set out to investigate correlations of DNA modifications as determined in this study to the mutations as observed in the genomes of our serogroup A strain collection [29]. The Single Nucleotide Polymorphisms were determined based on the genome sequence of strain Z2491 as reference and presumingly reflect the *in vivo* mutation and selection processes within the bacterial population associated with the meningitis epidemics. These settings contrast to classical experiments assessing effects of DNA methylation on nucleotide changes in *E*. *coli* evolving under laboratory conditions [47]. For the case of *N*. *meningitidis*, homologous recombination contributes significantly to sequence diversity [48], causing a potentially large fraction of the observed nucleotide changes.

From the total number of 6031 SNPs filtered for repeats and for recombinant fragments in the genomes of these strains and from the 20537 methylated nucleotides based on the consistent DNA methylation target sequences (AC^6m^ACC, C^5m^CGG, Y^5m^CTGG, GGNN^5m^CC) we would expect from a random distribution a total of 6031 SNPs /1.6Mb * 20537bp = ~77 SNPs occurring per chance on a methylation target site in a 1.6Mb repeat-excluded genome length. We actually observed a total number of 201 SNPs overlapping a methylation site, representing a 2.6 fold over-representation. This global approach indicated that methylated nucleotides indeed have an increased likelihood of mutation in settings with *in vivo* mutation and selection processes. The corresponding 201 methylation sites detected in the Z2491 genome did lose their function as target sites by the occurrence of the SNPs in the sequences of our serogroup A strain collection.

**Fig 6:**
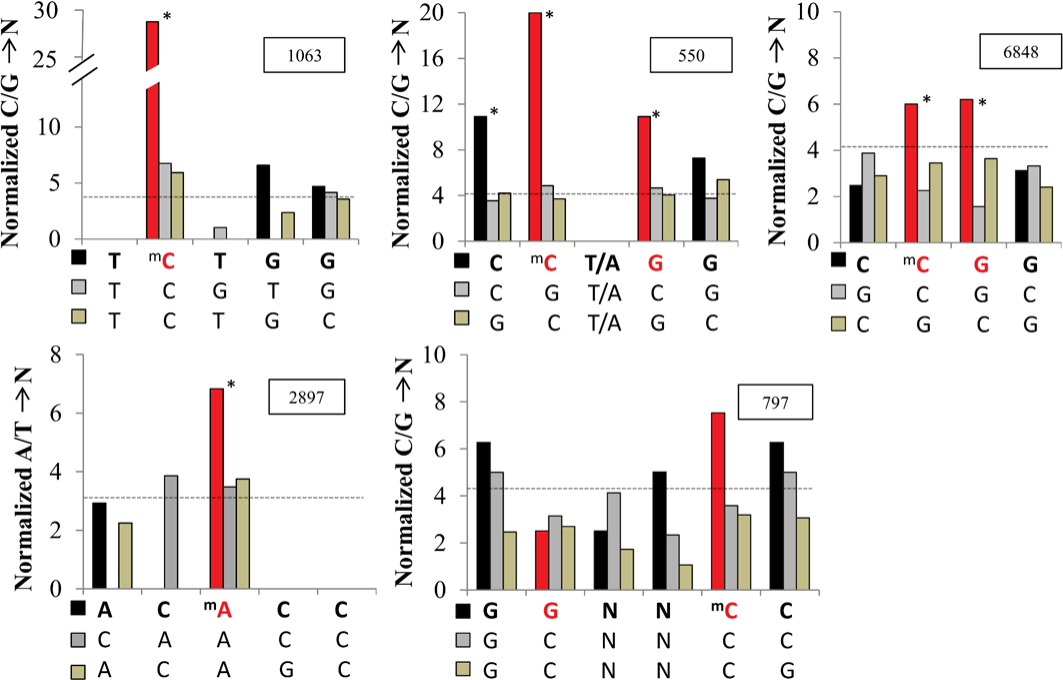
Methylated nucleotides display higher mutation rates than non-methylated positions. Positional co-occurrences of (in total 6031) SNPs at positions within (methylation) target motifs. Methylated positions highlighted in red within five target motifs (bold), as detected in the present study, with, each compared to two similar control sequences. Black bars in histogram represent nucleotides in methylated motifs, gray shades represent sequences not known as DNA-methylation targets. For each motif, counts of overlapping SNPs (for 5m-cytosine motifs: C/G in reference →N; or for 6m-adenosine: A/T→N) at each position are normalized by the genome-wide motif occurrences (numbers for methylated motifs in inset box). The dashed lines indicate the corresponding number of SNPs expected from random occurrence (G/C or A/T) across the genome and over-representation was tested with the χ2 statistics (*p value < 10–5).

To highlight the specificity of this effect to the methylated base position, we assessed the average number of SNPs at each motif position, normalized by the number of genome-wide occurrences of the motif. Given that our SNP calling could not determine the strand affected by a mutation, we considered both complementary bases. Fig 6 represents five of the methylation target motifs detected in this study. We compared the SNP counts (C/G→N or A/T → N) at each position of methylated motifs, or of scrambled non-methylated sequence motifs. The cytosine positions (T^5m^ **C**TGG, C^5m^ **C**WGG) consistently methylated in both strains as well as the methylated adenosines in the phase variable AC^6m^ **A**CC target motif displayed a ~2–3 fold significantly higher co-occurrence rate of SNPs, if compared to corresponding positions within scrambled motifs with unmethylated bases (p value < 10^−5^). While mutability at 5mC methylated nucleotides was already described, a mutational hotspot at 6mA methylated nucleotides has not been reported before. Non-methylated nucleotides in neighboring positions within the same motif, or within motifs not identified as methylation targets featured SNP occurrence rates close to the expected overlap if assuming randomly distributed SNPs. SNP classes (synonymous, non-synonymous, intergenic) might reveal divergent selective pressures, we did however not observe significant differences for SNPs overlapping methylated bases (S2 Fig). The target motif ATGC^6m^AT detected in this study displayed a tendency to increased co-occurrence rates with SNPs at methylated positions, however the motif was excluded due to a low number of only 128 occurrences in the Z2491 genome. For palindromic motifs, to avoid counting of SNPs double on both the forward and the reverse strand, we considered only the matching position on the forward strand. Consistent with a full methylation on both strands the palindromic motifs C^5m^CGG and C^5m^CWGG showed a mirroring peak in SNP occurrence rates at the guanosine in the third or forth motif position, respectively, which correspond to the methyl-cytosine on the reverse strand. The methylated positions in the palindromic motif GGNN^5m^CC displayed a barely increased overlap with SNPs. The corresponding methyltransferase (M.NmeAORF1453P) is only active in strain Z2491 (Fig 4, Table 1). From the uniform inactivation of this methyltransferase in all our isolates by an identical premature stop mutation we can assume an early time point of this mutation event in the evolutionary history separating our genomes from a common ancestor genome. Therefore the limited overlap of SNPs is consistent with a loss of methylation at GGNNCC, further supporting mutation rates depending on the duration of DNA methylation during evolution of the genomes.

## Discussion

We applied SMRT sequencing to genomes of the facultative human pathogen *Neisseria meningitidis*. The thereby determined DNA modification profiles of related isolates revealed similarities and differences in DNA methylation motifs, which could be associated with the presence of intact ORFs of a set of methyltransferase genes. Part of the differential DNA methylation could be attributed to the phase variable state of corresponding DNA methyltransferase genes. We furthermore assessed the positional distribution of the detected methylation target motifs within the genome assemblies. Clear occurrence biases of methylation motifs within presumable regulatory sequences are suggesting a role of DNA methylation in gene regulation, possibly related to proposed differences in antimicrobial susceptibility [49]. As introduced above, reported associations of DNA methylation in prokaryotes with timing of DNA replication, chromosome partitioning, DNA repair, and regulation of gene expression mostly still lack mechanistic evidences. In contrast to studies in other organisms [24] [17] we do not know the precise genomic coordinates of the transcription start sites (TSS) in *Neisseria*, but DNA methylation even within 5'UTRs could hypothetically affect interactions with DNA binding proteins or directly modulate RNA polymerase processivity (alike the effects exploited in SMRT sequencing). In contrast to the generalized decrease of 5mC motifs, we observed an enrichment of the 6mA target motif ACACC in the immediate upstream region of genes, which might relate to an enrichment reported for two m6A methylated target sites near the TSS [17]. Direct comparison of these approaches is however impaired by the afore mentioned divergent positional resolution. Moreover in our cross-sectional analysis of *Neisseria* isolates, we detected a prominent colocalization of SNPs with methylated bases, demonstrating an association of DNA methylation with mutagenesis and the evolution of genomes. This may have general implications for other prokaryotic populations.

### Detection limits for 5mC modifications

Our genomic DNA samples were subjected to an enzymatic conversion of 5mC to 5caC via the Tet1 enzyme. We detected kinetic signals for DNA methylation at only a fraction of the 5mC target sites in the genome. In contrast we observed a genome-wide complete protection of GGNN^5m^C**C** target sequences in a methylation-sensitive restriction digest. This apparent discrepancy is likely related to limitations in the detection sensitivity of 5mC modifications by the SMRT technology, as described previously [30].

Our data analysis procedures required some consistency in DNA modifications within the pool of individual cells subjected to SMRT sequencing. Consequently the absence of SMRT modification calls can not be interpreted as complete absence of any DNA modification at this locus. Conceptually intra-sample variations in DNA modifications could occur at different loci in the genome, for instance if some of the potential target sequences are 'masked' for the DNA modification enzymes by other DNA binding complexes. Such divergent modification patterns at specific loci reminding of eukaryotic cellular differentiation mechanisms have been previously observed [16], however only for modifications on adenosines bases, presumably due to the experimental limitations mentioned above.

At present potential biological variability can thus not be distinguished from the technical variability, specifically for 5mC. Further development of analysis methods might enable the detection of additional divergence in individual cells or at specific loci.

### Sequence variability in clonal populations

In addition to the differences between strains we also observe divergent repeat unit counts in genomic sequences extracted from clonal populations. Extending the considerations above, we do not observe such variability for all samples and for all loci, suggesting site-specific mutagenesis mechanisms. Albeit the sequencing coverages applied in these studies did yield in only a few reads with divergent repeat unit counts, we might nevertheless be detecting the products of phase variable mutations occurring during expansion of a clonal cell population. Verification of this hypothesis might require extreme precautions in preparations of genomic DNA samples and high-coverage sequencing in distant facilities to exclude the possibility of cross-contaminations of samples.

### Functional consequences of highly variable DNA modifications

Previous studies described the direct consequences of variable numbers of microsatellite repeat units within the ORF of two methyltransferase genes (modB2 and modA12) on the ORF status and consequently the DNA methylation profiles [21] [42] [18]. The rate at which these phase variability mutations occur and underlying mechanisms are incompletely characterized [46]. Reports describing mutations rates at other phase variable loci in *N*. *meningitidis* described drastic differences between serotypes, possibly linked to mismatch repair systems [50].

We do reveal in our study a non-random positioning of the methylation target sites which might suggest an involvement in gene regulation. Altered RNA abundance levels of a set of genes are however difficult to interpret as direct consequence of divergent global DNA methylation activities, as typically there is a very limited correlation of deregulated transcripts and DNA methylation target sequences observed [25]. Mutations in target sequences associated with divergent expression levels of specific genes could reveal more mechanistic insights. Our observation of increased numbers of SNPs precisely at methylation sites may however indicate that the regulatory mechanisms feature large degrees of plasticity and redundancy.

An additional consequence of the presence of restriction sites was reported on the DNA uptake sequence-dependent transformation [51]. Specific DNA methylation profiles in *N*. *meningitidis* strains might thus define 'compatibility groups' for horizontal DNA transfers within the microbial community in the human nasopharynx [52] [53] [6]. We could not identify significant biases in the presence or absence of DNA methylation target sites within putative recombinant fragments. These fragments putatively originating from *N*. *lactamica* or *N*. *gonorrhoeae* constituted about 20% of the genome length, and are containing a matching proportion of methylated motifs.

In the present study we detected a significant enrichment of SNPs between genomes of *N*. *meningitidis* serogroup A strains at positions of methylated bases within DNA methylation target motifs. The causes of this correlation and the consequences on genome evolution are at present not clear. Replication-transcription conflicts were associated with higher mutation rates [54]. Therefore we determined leading and lagging strands based on the annotation of the origin of replication in the Z2491 genome. Strikingly, of the 4096 instances of the non-palindromic AC^6m^ACC target motifs within the genome of the Z2491 strain, only 33% occur on the leading strand, which might relate to the observation of a clear bias (60.2%) of ORFs on the leading strand in one replichore [34]. Accordingly the observed biased distributions of specific DNA methylation target motifs might be either the consequence of increased mutations at these sites or represent selection pressure to exclude or maintain DNA methylations sites at intergenic regulatory regions. Strikingly we could not discern major biases, SNPs overlapping methylated nucleotides showed a similar distribution between intergenic and coding regions. Similarly no bias was observed between synonymous and non-synonymous SNPs within the coding region deviating from the overall SNPs segregation (S2 Fig). Therefore we conclude that selective pressures are similar on mutations associated with DNA methylation.

Recent comparative genome analysis has considerably expanded our knowledge of prokaryotic defense systems [55]. Specifically the presence of apparent conflicts between restriction systems [56], or orphan methyltransferases lacking cognate restriction enzymes [57] hint for more complex biological roles of prokaryotic DNA methylation. The precise effect of DNA methylation on mutation rates in prokaryotes is presently unclear due to multiple levels of mutational, mismatch repair, and selection mechanisms [58]. The damage of an uracil base resulting from deamination of an unmodified cytosines can typically be corrected [59]. Original studies describe an increased rate of spontaneous deamination of 5-methylcytosine compared to cytosine residues [60]. Deamination of 5-methylcytosine results in a 'genuine' thymine base. In the context of double stranded DNA molecules, mismatch repair mechanisms have therefore limited means to detect and repair unequivocally the newly mutated nucleotide in a G/T mismatched pair. Repair systems counteracting the mutagenic effects of hydrolytic deamination of 5mC (Vsr endonucleases) have been described in *N*. *gonorrhoeae* [61], yet we have no evidence for activities of orthologous genes (V.NmeIP) in *N*. *meningitidis*. Methylated bases have been reported to be mutational hotspots for instance in mutation-accumulation studies in *E*. *coli* in laboratory settings [47]. Our present study addresses for the first time the association of experimentally confirmed DNA methylation and the genome evolution in an *in vivo* setting. Here a number of additional processes are involved in the selection of favorable configurations of genome structures at different scales [62]. Our results suggest that DNA methylation and evolutionary processes are two processes intimately correlated. Despite the highly variable activities of DNA methyltransferase genes in evolutionary timescales, genomic and epigenomic factors contribute in a complex interplay to the evolution of the optimally adapted prokaryotic populations.

## Conclusions

SMRT sequencing determines DNA methylation profiles of prokaryotes at a genome-wide level.

This study contributes to the recognition of a previously underestimated potential for variability in DNA methylation. The discovery of biased presence of methylation target motifs in genomic sequences may indicate a role in gene regulation. The increased occurrences of mutations precisely at methylation target positions suggests additional yet unidentified functional consequences of DNA methylation and Restriction-Modification (RM) systems in the evolution of prokaryotic genomes.

## Supporting Information

S1 Fig SMRT sequencing on samples without Tet1 conversion detects modified adenosines DNA modification scores are plotted against the coverage in SMRT sequencing of samples. Each dot represents a position on either strand with a modification score larger than 20, the color specifying the nucleotide base, on which the modification was detected. Modified adenosines (red dots) are predominantly detected in strain NM1264. The horizontal line indicates the threshold score 50 applied for subsequent motif finding. (See Fig 1 for Tet1 converted samples)
(TIF)

S2 Fig Comparable distribution of SNPs overlapping methylated bases: 6031 SNPs as observed within the repeat-filtered genome assemblies of a strain collection of *N*. *meningitidis* are classified into synonymous and non-synonymous mutations in coding sequences, or attributed to intergenic. SNPs overlapping methylated bases display a very similar distribution, indicating that selective pressures are similar on mutations associated with DNA methylation.
(TIF)

S1 Text Table in gff format specifying 198 sequence variants SMRT vs. Illumina: Aliquots of the same genomic DNA preparation of strain NM1264 were subjected to sequencing by either the Illumina or by the SMRT sequencing method. (GFF)

S2 Text SMRT sequencing modification scores *Neisseria meningitidis* strain Z2491: gff file specifying genomic coordinates of positions with SMRT DNA modification scores larger than 20 (column 6) (GFF)

S3 Text SMRT sequencing modification scores *Neisseria meningitidis* strain NM1264: gff file specifying genomic coordinates of positions with SMRT DNA modification scores larger than 20 (column 6) (GFF)

S4 Text Partial genome assembly of *Neisseria meningitidis* strain NM1264: 344 contigs in multi-fasta format, assembled from Illumina reads, ordered according assembly of reference strain Z2491 (FA)
