# Supplementary figures and images for: DNA Methylation Assessed by SMRT Sequencing Is Linked to Mutations in Neisseria meningitidis Isolates
Source: PLoS One. 2015 Dec 11;10(12):e0144612. doi: 10.1371/journal.pone.0144612 (PMC4676702; doi:10.1371/journal.pone.0144612)

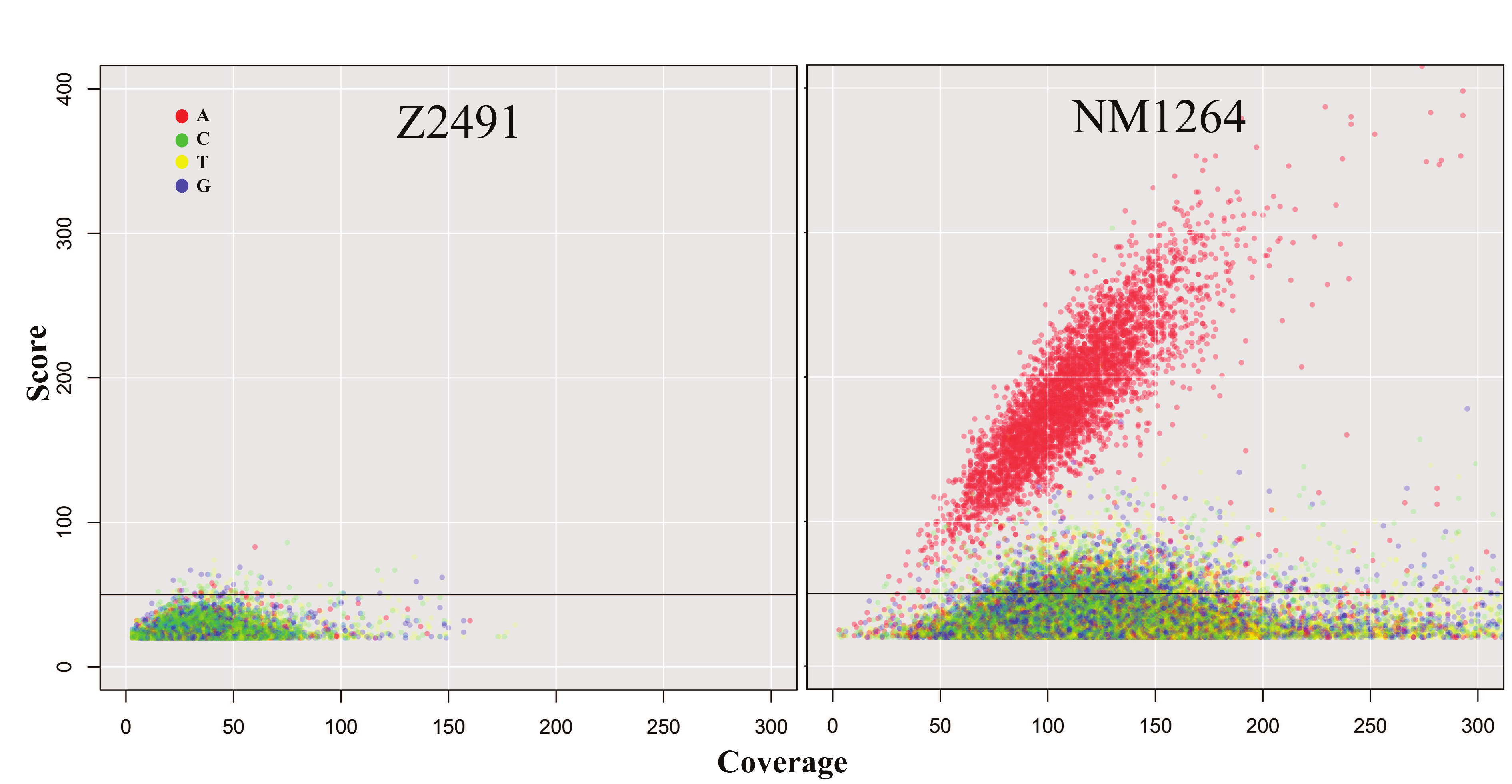

Supplement: S1 Fig — Each dot represents a position on either strand with a modification score larger than 20, the color specifying the nucleotide base, on which the modification was detected. Modified adenosines (red dots) are predominantly detected in strain NM1264. The horizontal line indicates the threshold score 50 applied for subsequent motif finding. (See Fig 1 for Tet1 converted samples) (TIF) [file pone.0144612.s001.tif]

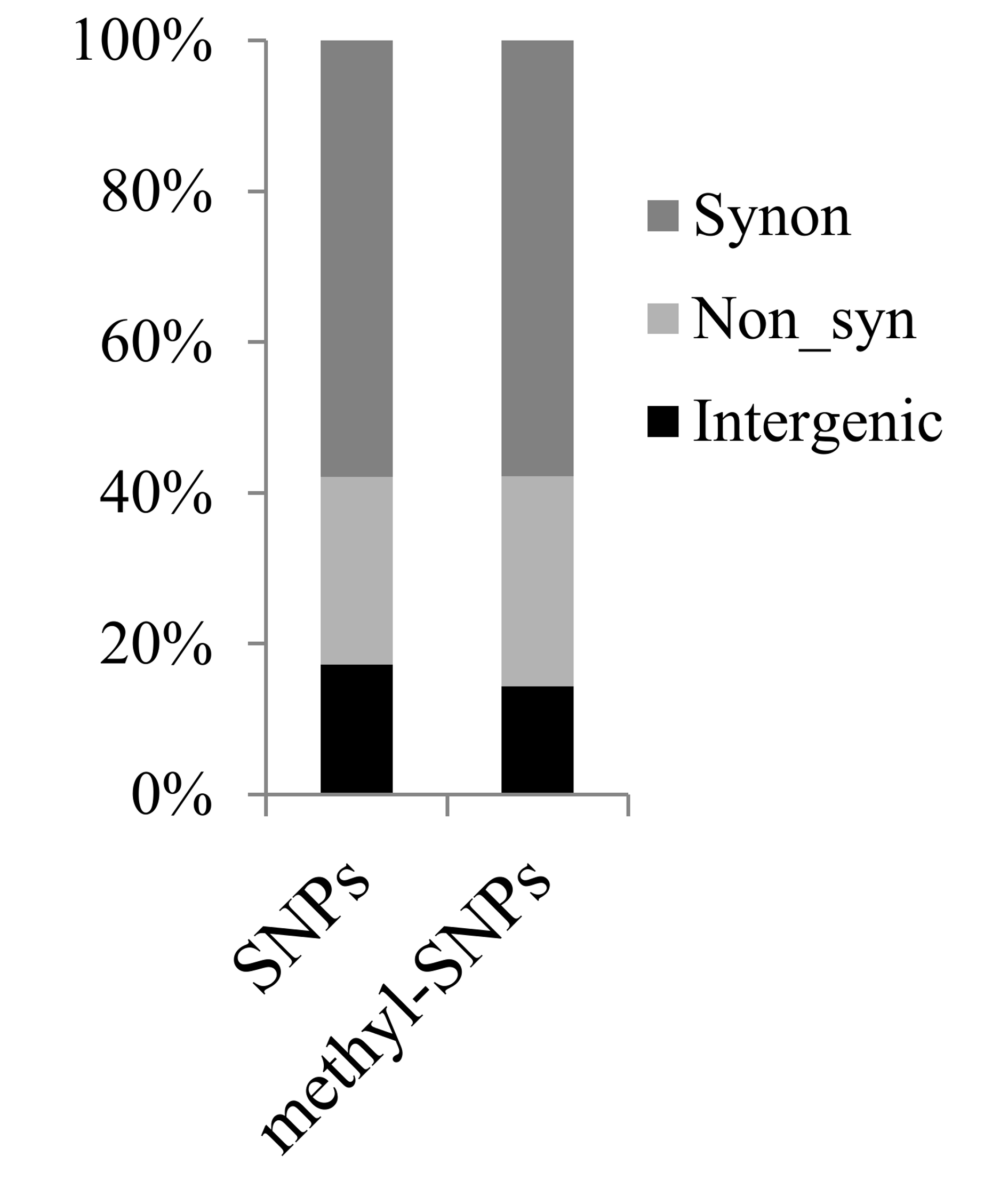

Supplement: S2 Fig — SNPs overlapping methylated bases display a very similar distribution, indicating that selective pressures are similar on mutations associated with DNA methylation. (TIF) [file pone.0144612.s002.tif]
